# Dissolution of Amorphous S53P4 Glass Scaffolds in Dynamic In Vitro Conditions

**DOI:** 10.3390/ma14174834

**Published:** 2021-08-26

**Authors:** Laura Aalto-Setälä, Peter Uppstu, Polina Sinitsyna, Nina C. Lindfors, Leena Hupa

**Affiliations:** 1 Johan Gadolin Process Chemistry Centre, Åbo Akademi University, Henrikinkatu 2, 20500 Turku, Finland; laura.aalto-setala@lasifaasi.fi (L.A.-S.); polina.sinitsyna@abo.fi (P.S.); 2 Polymer Technology Research Group, Faculty of Science and Engineering, Åbo Akademi University, Henrikinkatu 2, 20500 Turku, Finland; peter.uppstu@abo.fi; 3 Department of Musculoskeletal and Plastic Surgery, Helsinki University Hospital, PL 3 00014 University of Helsinki, 00260 Helsinki, Finland; nina.c.lindfors@hus.fi

**Keywords:** bioactive glasses, S53P4, glass scaffolds, sintering, in vitro dissolution, dynamic dissolution, biomaterials, tissue engineering

## Abstract

The silicate-based bioactive glass S53P4 is clinically used in bone regenerative applications in granule form. However, utilization of the glass in scaffold form has been limited by the high tendency of the glass to crystallize during sintering. Here, careful optimization of sintering parameters enabled the manufacture of porous amorphous S53P4 scaffolds with a strength high enough for surgical procedures in bone applications (5 MPa). Sintering was conducted in a laboratory furnace for times ranging from 25 to 300 min at 630 °C, i.e., narrowly below the commencement of the crystallization. The phase composition of the scaffolds was verified with XRD, and the ion release was tested in vitro and compared with granules in continuous flow of Tris buffer and simulated body fluid (SBF). The amorphous, porous S53P4 scaffolds present the possibility of using the glass composition in a wider range of applications.

## 1. Introduction

Bioactive glasses (BAGs) have been a subject of intense research since their discovery by Hench et al. in 1971 [1]. In the first clinical applications, the glasses were used as granules or small monoliths [2]. In the past 10 years, the research focus has shifted towards developing porous scaffolds based on BAGs for load-bearing bones, as summarized in several review papers [3,4,5,6,7]. The load-bearing property is approached either by using BAGs as a non-load-bearing part of composite scaffolds [8,9,10,11,12] or by sintering the glass into mechanically durable structures using different techniques [13,14,15].

Recently, the BAG research has broadened the scope beyond bone tissue treatments: BAGs have been shown to be able to accelerate soft tissue healing, including damaged skin, lung tissue and nerve tissue [16,17,18,19]. BAGs, including S53P4, have also shown strong antibacterial effects [20,21]. BAGs can stimulate cell proliferation and angiogenesis [22,23] and reduce inflammation of injured sites [18]. Any advances in preparing scaffolds, initially aimed for bone regenerating applications, will also aid the development of scaffolds for soft tissue applications.

There is an active field of research towards manufacturing BAG scaffolds using various methods, including foam replication, additive manufacturing and laser sintering [24,25,26,27,28]. In addition, sol–gel techniques enable manufacturing mesoporous BAGs, which can be used in targeted drug delivery, providing controlled release of, e.g., neural drugs [29,30,31,32]. The different techniques allow different pore structures, but they cannot overcome the inherent brittleness of glasses. In order to improve the mechanical properties, there has been a growing interest in using polymers as reinforcement materials [10,11,27,33]. The bioactivity and antibacterial effects of BAGs can also be utilized by applying BAGs as coatings, for which the modern techniques range from laser cladding to electrochemical deposition [34,35,36,37].

The silica-based BAGs 45S5 and S53P4 are U.S. Food and Drug Administration (FDA) approved for specific clinical bone restorative applications in granule or particle forms [2,38,39,40]. However, these two BAGs easily crystallize when their particles are thermally treated in the viscosity range for achieving porous tissue engineering scaffolds through viscous-flow sintering [41]. Glass 45S5 crystallizes shortly above the glass transition temperature before its melt has reached a sufficiently low viscosity level required for viscous-flow sintering. This composition thus cannot be sintered into strong, load-bearing amorphous scaffolds without crystallization. Porous, strong and highly crystalline 45S5 glass-ceramic scaffolds have been manufactured through thermal treatment at around 900–1000 °C, i.e., well above the glass transition temperature of BAG 45S5 [25,42,43]. Glass S53P4 has a somewhat broader sintering temperature window, enabling the manufacture of amorphous or partly crystalline scaffolds in carefully controlled conditions [44]. Fagerlund et al. have reported amorphous S53P4 scaffolds sintered at 635 °C that had a low mechanical durability (0.7 MPa). The strength increased at higher temperatures (up to 10 MPa at 1000 °C), together with sodium–calcium–silicon crystals starting to form at particle surfaces. The content of the primary crystals varied with the heating rate and the sintering temperature [44].

If the BAG crystallizes during sintering, the obtained scaffold is not purely amorphous but a glass-ceramic, consisting of one or more crystalline and residual amorphous phases. The dissolution of glass-ceramic 45S5 is slower than that of the parent glass [43,44,45]. As crystallization alters bioactivity [45], the produced scaffolds should remain amorphous for maintaining the verified properties of the parent BAG to be regarded as different products of the same material. Over the years, several BAG compositions with lower crystallization tendency have been tailored to better suit hot working [15,46,47,48,49,50], and scaffolds strong enough for load-bearing or semi-load-bearing applications have been produced from some tailored compositions [13,14,15,51]. 

As even small changes in composition can lead to differences in dissolution behavior [52], the biological response might also change. Changes to BAG compositions thus require extensive additional preclinical and clinical trials for regulatory approval. Ideally, scaffolds for implantation are produced from a BAG already approved for bone-grafting applications.

In this study, we report optimizing the sintering conditions for porous, amorphous scaffolds of the BAG S53P4 in a laboratory furnace. The properties essential for in vivo implantation were mimicked using long-term in vitro dissolution experiments in continuous flows of simulated body fluid (SBF) and Tris buffer solutions. The scaffold porosity was consistent with a previous in vivo study, in which the partially crystallized S53P4 scaffolds allowed bone ingrowth but were very fragile [53]. Although the sintering parameters suggested are valid for a laboratory furnace arrangement, the results provide guidelines for developing the sintering of amorphous S53P4 scaffolds for pilot and full-scale manufacturing processes.

## 2. Materials and Methods

### 2.1. Glass Scaffold Preparation

**Figure 1:**
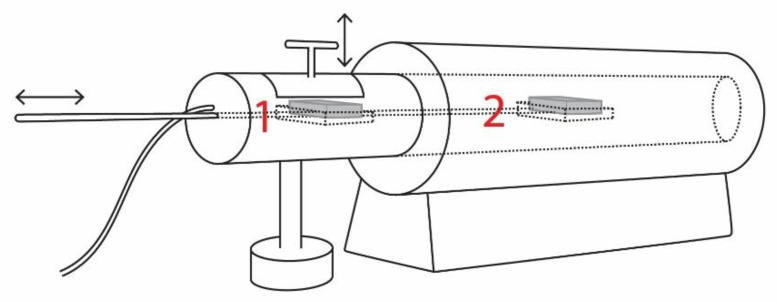
Schematic image of the in-house modified Carbolite furnace CTF/12/65 used for sintering. In the setup, the lid is opened and graphite mold with glass is first placed in a holder in a nitrogen-filled, nonheated chamber (1) before the holder is pushed to the middle of the furnace for sintering (2). Image not to scale.

Melt-derived BAG S53P4 (53.9% SiO_2_, 22.7% Na_2_O, 21.8% CaO and 1.7% P_2_O_5_; all in mol.%) granules of the size fraction 315–500 μm were kindly provided by BonAlive Biomaterials Ltd. The granules were sintered to porous scaffolds at 630 °C in graphite molds in nitrogen in an electric Carbolite tube furnace (model CTF/12/65) with some in-house adjustments (Figure 1). The sample loaded in the graphite mold was placed on a tray in a chamber outside the furnace opening door. After filling the chamber with nitrogen, the sample was pushed using the sliding tray into the furnace at the sintering temperature. Nitrogen was constantly fed into the furnace to protect the mold, and the sample temperature was recorded using a K-type thermocouple in the mold. The sintering time recording started when the sample was pushed to the middle of the furnace. After sintering, the tray was pulled back to the outer chamber now used for cooling the sample in nitrogen.

The sintering temperature corresponded to the onset of crystallization for BAG S53P4 according to thermodynamic analysis [54]. Fifteen different sintering times, ranging from 25 to 540 min, were used to achieve strong amorphous scaffolds. The sintering was conducted to yield cylindrical scaffolds of the following two different sizes: (i) diameter 9.4 mm and height 10.0 mm (mass 826 ± 26 mg) for the compression studies and (ii) diameter 5 mm and height 10 mm (mass 272 ± 6 mg) for the dissolution studies.

Partially crystalline cylindrical scaffolds (diameter 9.7 mm, height 10.0 mm) were used as references in the compression tests. These scaffolds were sintered using the same furnace setup as in the previous study, namely 720 °C for 90 min [53] in a front-loading box-type Nabertherm oven.

### 2.2. Phase Composition

The amorphous nature of the scaffolds after different sintering times was studied using an Empyrean X-ray diffractometer (Malvern Panalytical, Almelo, The Netherlands, Cu α radiation, 40 mA, 40 kV, 10–80° 2θ, 2.0°/min). The scaffolds were powdered with an agate mortar and pestle before the analysis.

### 2.3. Porosity

The porosity was determined from five cross-sectional SEM images of 30× magnification for each of the scaffold types (sintered for 25 to 540 min) from the SEM images using Photoshop CS6 software (Adobe Systems Inc., San Jose, CA, USA). The mass and dimensions of the scaffolds were also measured and compared to the theoretical mass of a solid glass S53P4 cylinder of the same size. When calculating the porosity, the reported density of 2.66 kg/dm^3^ for S53P4 was used [55].

### 2.4. Dissolution Tests

The dissolution of freely packed granules and scaffolds sintered for 60 min (a time chosen based on the XRD and porosity measurements) was studied in a continuous flow reactor cell developed by Fagerlund et al. [56]. Fresh SBF or Tris was fed longitudinally through the glass sample in the reactor cell using a flow rate of 0.20 mL/min. Both the solution and the reactor cell were kept at 37 °C.

SBF was prepared according to the protocol developed by Kokubo et al. [57]. Tris solution (50 mM, Trizma base, Sigma-Aldrich, Burlington, MA, USA was adjusted with 1 M HCl (J.T. Baker, Phillipsburg, NJ, USA) to pH 7.40 at 37 °C. Two dissolution experiments with sintered scaffolds were conducted in both solutions, along with one experiment using granules of S53P4 in each solution as a reference. The masses of the scaffolds in Tris solution weighed 276.6 and 271.9 mg; scaffolds in SBF solution weighed 272.5 and 265.5 mg. The sample mass in both granule experiments was 233 mg. Both the scaffolds and granules filled the same volume inside the reactor cell; the weight difference was due to granules packing looser compared to sintered scaffolds. The scaffolds were weighed dry before and after the experiments.

The in vitro solutions were fed through the BAG scaffolds and granules for 7 days. On the first day, solution fed through the reactor cell was collected for three hours, and samples were taken and analyzed.

Three subsequent short-interval (15 min) samples of the solutions were collected daily after the solution had passed the reactor containing the glass scaffold. The concentrations of the ions dissolved from the glasses into the solution were analyzed using an inductively coupled plasma optical emission spectrometer (ICP-OES, Optima 5300 DV; Perkin Elmer, Waltham, MA, USA). Before the analysis, the solutions were diluted with ultrapure water to a ratio of 1:9. The elements analyzed were silicon (λ = 251.611 nm), calcium (λ = 317.933 nm) and sodium (λ = 589.592 nm). Phosphorous levels were close to the limit of quantification (LOQ) and are thus not reported. The calibration was conducted using ultrapure water and multielement standard (Multi-Element Standard 25, Perkin-Elmer, Waltham, MA, USA) and silicon standard (Ultra Scientific, North Kingstown, RI, USA) with 1 ppm concentrations of Ca, Na, K and Si. The calibration was rechecked after every 20 solution samples. All reported values are background corrected.

### 2.5. Analysis of the Glass Surfaces

After the in vitro experiments, the remaining scaffold pieces and glass granules were washed in ethanol, dried, weighed, cast to polyester resin and polished to reveal the surface layers that had formed during the dissolution tests. These reaction layers were studied using a Leo Gemini 1530 Scanning Electron Microscope, SEM (Carl Zeiss, Oberkochen, Germany). An UltraDry X-ray detector (Thermo Fisher Scientific, MA, USA) was used for the EDX analyses of the layer compositions. 

### 2.6. Compression Tests

The compressive strength of the scaffolds sintered for 60 min and the partially crystallized reference scaffolds were tested using Lloyd LR30K Plus Materials Testing Machine (Lloyd Instruments, Bognor Regis, UK). The rate of compression was 1 mm min^−1^ up to 3 mm of compression. Five scaffolds were tested dry and five scaffolds were tested in a wet state after 5 min of static immersion in SBF.

## 3. Results

### 3.1. Phase Composition

**Figure 2:**
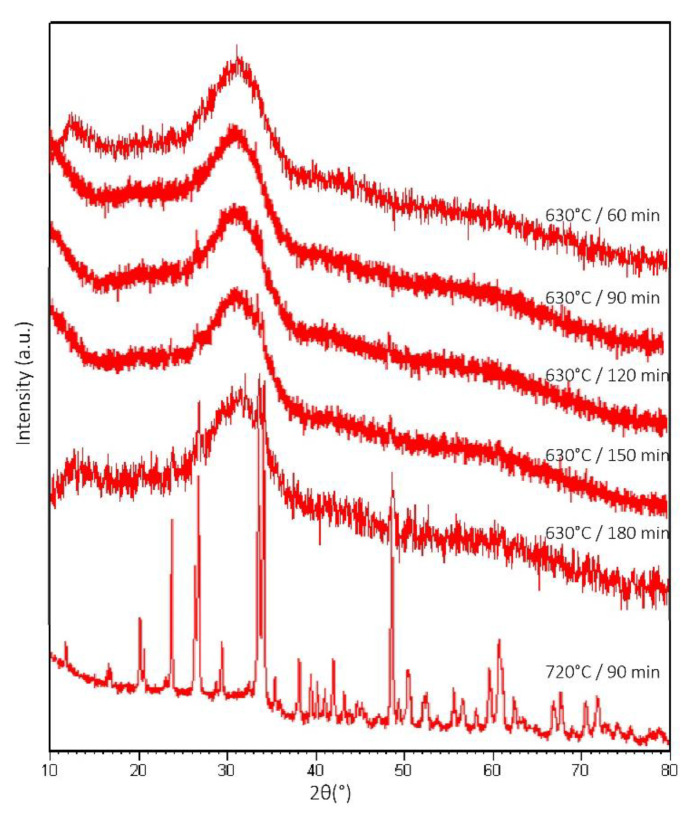
XRD analysis of S53P4 scaffolds sintered for 60 to 180 min at 630 °C and 90 min at 720 °C. A.U. refers to arbitrary units.

Figure 2 shows the diffractograms of the scaffolds sintered at 630 °C for 60 to 180 min. No distinct signs of crystallization were detected for scaffolds sintered for 60 min. However, after longer sintering times, peaks indicating crystallization of combeite (Na_2_CaSi_3_O_8_) were observed. The reference scaffold sintered at 720 °C showed distinct peaks of combeite.

### 3.2. Porosity

The porosity and the possible presence of crystalline phases in the scaffolds sintered for 25 to 540 min 630 °C were determined from SEM images. In an in vivo study of S53P4 scaffolds sintered at 720 °C for 90 min, a total porosity of 49 ± 2% provided the desired tissue ingrowth [58]. In this work, a similar overall porosity level, 50 ± 3%, was achieved for scaffolds sintered for 60 min at 630 °C. For the longer sintering times, the porosity was slightly lower (data not shown as there were signs of crystallization for sintering times over 60 min). The calculated porosity was 54%, based on the dimensions and mass of the amorphous scaffolds sintered for 60 min at 630 °C. 

### 3.3. Ion Concentrations

**Figure 3:**
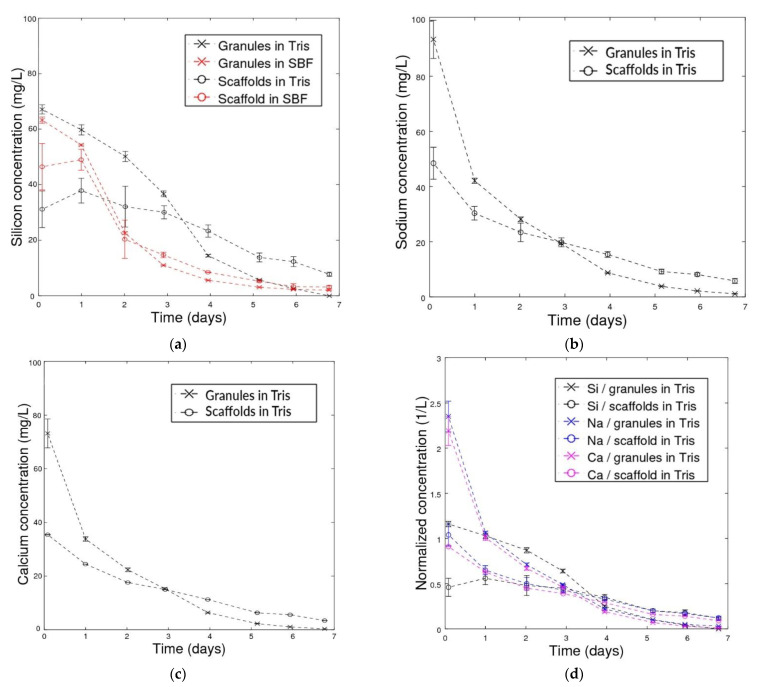
Ion concentrations dissolving from S53P4 granules and amorphous scaffolds under a continuous flow of Tris and SBF, in (**a**) silicon in Tris and SBF, (**b**) sodium in Tris and (**c**) calcium in Tris. In (**d**), concentrations released into the continuous flow of Tris as functions of time were normalized with respect to the amount of each element in the glass.

Concentrations of silicon, sodium and calcium ion species dissolving from the glasses in the continuous flow of Tris or SBF as functions of experimental time are shown in Figure 3a–c. Although all concentrations were higher initially for granules than for scaffolds, the granules released smaller concentrations than scaffolds after the first 2–4 days. In SBF, the silicon species dissolved at almost identical rates from the granules and scaffolds after a notable difference in the first days. For granules in Tris, the concentrations of all ion species decreased to low values at 7 days. For scaffolds, the ion concentrations gradually decreased but at a slower rate than for the granules. To gain further insight into the release of the ions, their concentrations were normalized over the molar amount of each ion present in the nominal glass composition (Figure 3d). For times longer than 1 day, the normalized ion concentrations were approximately at the same level, thus indicating that their release into Tris was congruent at longer time points.

**Figure 4:**
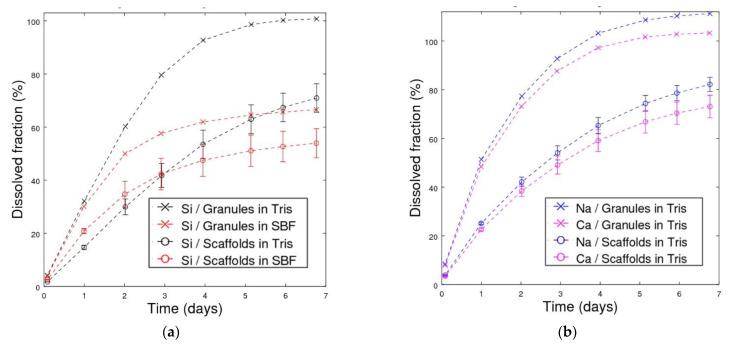
Dissolved fractions of (**a**) silicon and (**b**) sodium and calcium, presented as cumulative values estimated from the measured short-interval measurements. The dissolved fraction is the percentage of dissolved Si compared to the initial amount of Si in the studied specimen (for glass S53P4, there is 25 mass% of silicon).

The calculated cumulative silicon species dissolution is shown in Figure 4a. The ion release was assumed linear between two subsequent time points. The cumulative dissolution of silicon ions was similar in Tris and SBF after 1 day, but after 2 days and onwards, the dissolution in SBF slowed down, and only 67% of silicon had dissolved after 7 days. In contrast, all silicon had dissolved from granules in Tris after 7 days. Similarly, for scaffolds in Tris and SBF, the cumulative dissolution of silicon in Tris after 7 days was 71%, whereas it was estimated to be 54% in SBF.

### 3.4. Mass Loss

The granule and scaffold masses before and after the dissolution tests are listed in Table 1. Only approximately 1 wt% of the granule sample remained after 1 week in Tris, whereas the weighed mass loss was 52% after SBF dissolution. The mass loss was also considerably higher in Tris than in SBF for the scaffolds.

### 3.5. Reaction Layer Formation

**Figure 5:**
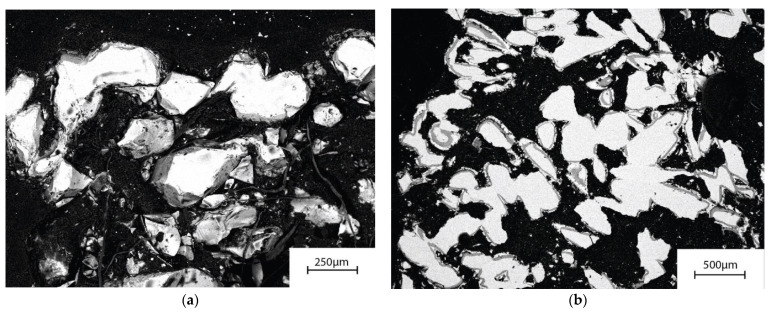
SEM images of cross-sections of an amorphous S53P4 scaffold after 7 days of the continuous (**a**) Tris and (**b**) SBF flow.

The amorphous scaffolds maintained their overall structure during the 7 days of the continuous flow of SBF and Tris. However, after dissolution in Tris, the scaffolds were extremely fragile and could not be prepared for SEM analysis without damaging the scaffold structure. Figure 5a shows a part of a scaffold after 7 days of Tris immersion. No signs of a calcium phosphate (CaP) layer were identified, but a silica-rich layer was analyzed on the fragment surfaces. The necks between the granules had decreased in thickness when compared with before dissolution. 

**Figure 6:**
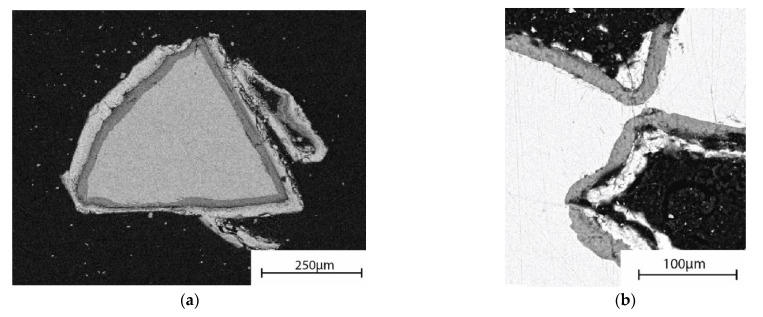
Detailed cross-sectional SEM images of amorphous S53P4 scaffold after 7 days of continuous SBF flow, highlighting (**a**) the different degree of degradation of granules of the same specimen and (**b**) how the CaP layer is reinforcing the necks of the scaffolds. The precipitated CaP layer (seen in white) is surrounding the silica-rich layer (dark grey) on the unreacted glass core (light grey/white).

In contrast to immersion in Tris, there was a distinct CaP layer on the glass surfaces after continuous flow of SBF (Figure 5b). Although the scaffolds had reacted to a large degree in the continuous flow of SBF, they were considerably stronger than those after Tris dissolution. This was also verified by the easy processing for the SEM analyses. There were CaP layer formations thickening the neck areas (Figure 6b). Overall, the degree of degradation varied considerably. Several specimen granules had evolved to CaP shreds with only remnants of attached silica-rich layer after 7 days (Figure 6a). Nevertheless, some granules from scaffold specimens showed a large amount of unreacted bulk glass surrounded by silica-rich and calcium phosphate rich surface layers (Figure 5b).

### 3.6. Compression Tests

**Figure 7:**
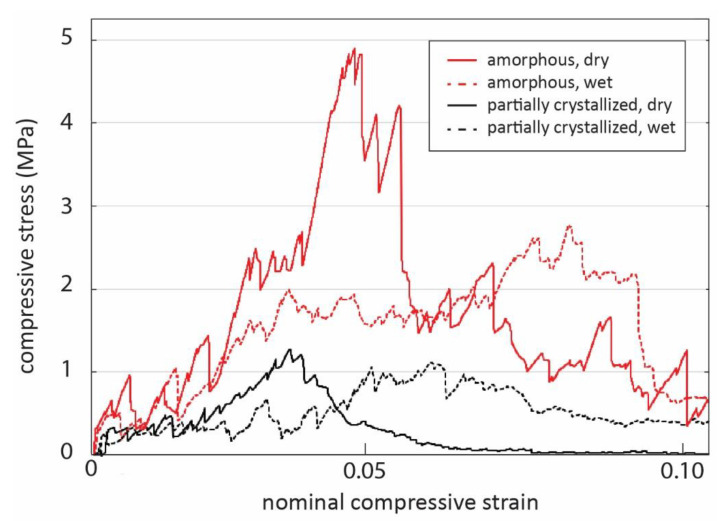
Representative stress–strain curves for amorphous scaffolds in compressive testing, with partially crystalline scaffolds as reference. The scaffolds that were tested in wet condition had been immersed in SBF for 5 min immediately prior to testing.

The results of compression tests are shown in Table 2. For the selected scaffolds (630 °C for 60 min) with 50% porosity, the compressive strength of the dry scaffolds was 4.8 MPa. The strength decreased markedly to approximately 2.9 MPa after 5 min of immersion in SBF. For the partially crystallized scaffolds of the same porosity level, the strength values were considerably lower (1.3 and 1.1 MPa in dry and wet conditions, respectively). Figure 7 shows representative stress–strain curves for amorphous and partially crystalline scaffolds in dry and wet conditions.

## 4. Discussion

The scaffolds sintered from BAG S53P4 at 630 °C for 60 min were amorphous and had a compressive strength of 5 MPa when dry, which enables handling and low-load in vivo applications. The values correspond to the compressive strength and porosity of cancellous bone, which are reported to be in the ranges of 2–12 MPa and 50–90%, whereas for cortical bone, the values vary in the ranges 100–150 MPa and 5–10% [59]. 

On one hand, scaffolds sintered for longer times had similar or lower porosity, but as XRD indicated crystallization, longer sintering times were omitted from compressive testing. On the other hand, scaffolds sintered for shorter times than 60 min at 630 °C showed no signs of crystallization but had not sintered to a sufficient degree, which caused them to break easily under manual inspection.

Fabert et al. [15] compared the porosity and compressive strength of different amorphous borosilicate bioactive glass scaffolds sintered from particles. According to their results, the porosity is the determining parameter over glass composition for scaffold strength. Our results of 5 MPa for scaffolds with 50% porosity are consistent with their results. The enhanced strength of the amorphous scaffolds compared to the partially crystallized S53P4 scaffolds of the same porosity is assumed to be due to poor bonding between the residual amorphous phase and crystals. In addition, unaligned crystals between neighboring particles provide suitable fracture lines when force is applied.

The necks between the granules in the amorphous scaffolds partly degraded and decreased in thickness during in vitro immersion. In continuous flow of Tris, there was no precipitation of CaP surface layers, resulting in scaffolds losing their structure. In continuous flow of SBF, a CaP layer formed at the surfaces and grew in thickness, which contributed to increasing scaffold strength.

Both Tris and SBF buffers were selected for the dissolution tests to easier identify the dissolution mechanisms of the porous scaffolds. SBF solution mimics the in vivo conditions better [8], but the downside is its high Na and Ca concentrations. After the initial ion burst, the released Na and Ca concentrations from the scaffolds were too low to give reliable deviations from the SBF background. In contrast, Tris solution does not background disturbance: it does not contain any sodium, calcium, silicon or phosphorous species. Still, the changes in the scaffold surface morphology depend on the ion release from the scaffold itself and are assumed to correlate with the ion release trends before a CaP layer forms on the surface, motivating the use of Tris solution alongside SBF. The long-term dissolution in SBF showed similar trends to in vivo studies [60].

The absence of precipitation layers in Tris solution also allowed the scaffolds and granules to dissolve more rapidly. The difference in the Si species dissolution rates between the granules and scaffold in Tris can be explained by the larger surface area of the granules and easier flow of solution between the particles.

Generally, the well-known bioactive glasses 45S5 and S53P4 are considered to crystallize before they sinter to amorphous scaffolds strong enough for semi- or low-load-bearing applications [41]. No amorphous 45S5 scaffolds have been reported; for S53P4, the only reported amorphous scaffolds had compressive strength of only 0.7 MPa [44]. 

During sintering of glass-ceramic scaffolds, the parent glass transforms into crystals and residual glassy phases, thus changing the dissolution behavior compared to the parent glass [44,54]. The size of the crystallites and their composition and share in the final product strongly depends on the sintering parameters. Thus, totally or partially crystallized 45S5 and S53P4 have different dissolution and reaction behavior compared to the parent glasses and crystalline samples sintered in other conditions. In contrast, the dissolution behavior of the amorphous scaffolds depends on the in vitro and in vivo conditions, e.g., local saturation of fluid, fluid flow rate and surface area glass-to-volume ratio.

The measured mass losses of the scaffolds in Tris were approximately 70% (Table 2), whereas the cumulative mass loss calculated from the released ion concentrations for silicon was 71%. In SBF, the measured mass losses were 40–50%, whereas the cumulative estimation was 54% dissolution of silicon. The measured mass loss correlated well to the calculated cumulative silicon release. A somewhat higher difference was seen for the Si release from the granules to Tris and SBF, corresponding to 99 wt% and 100% Si in Tris compared to 52 wt% and 59% Si in SBF (Table 2 and Figure 5a). This was assumed to be due to extensive CaP precipitation, which added mass to the specimens. XRD analysis of the scaffolds after the immersion was not conducted due to the small amount of the material left. The hydroxyapatite formation of BAG S53P4, when exposed to SBF, is well-established [56]. The SEM results in this work are in line with the published results.

For both granules and scaffolds, the Si release first increased rapidly but then slowed down as the CaP layer that formed in SBF started to prevent release into the solution. Although the dissolution of bioactive glasses has traditionally been assumed to be controlled by diffusion [61], there has recently been growing discussion on the topic. Based on the activation energies, Elgayar et al. [62] concluded that ion exchange is not the rate-limiting step in the dissolution of BAGs.

To assess whether the dissolution of the glasses is controlled by diffusion or chemical reaction rates, the cumulative dissolved silicon ion data were fitted to reaction rate controlling and diffusion controlled shrinking particles using shrinking core models [63]. The models assume spherical particles with a constant thickness of a diffusive barrier [63]. In fact, the reaction rate-controlled model predictions seemed to give a better fit for the Tris experiments. As the models do not consider the effect of the growing CaP precipitation layer, both models fail to describe the dissolution in SBF after the first days when the CaP has formed a partly protective surface layer.

BAG-based scaffolds are likely to react in vivo according to the steps suggested in the literature [64,65]. The dissolution begins with the development of a silica-rich surface layer due to alkali and alkaline earth ion exchange with hydrogen ions in the body fluid, as suggested by the ion release in Tris (Figure 3). After that, amorphous calcium phosphate starts to precipitate on the silica-rich layer already after a few hours, as seen in the dissolution studies in SBF. Although not verified in this work, the calcium phosphate layer gradually forms hydroxyapatite crystals similar to bone apatite [66]. In general, the overall porosity and pore size affect vascularized bone tissue formation throughout the scaffold, along with the scaffold in vivo degradation. Although the overall porosity of the scaffolds in this work was less than, e.g., in robocasted and template sintered bioactive glass scaffolds [7], similar S53P4 scaffolds showed stable scaffold integration after 8 weeks in rabbit femurs [60].

The evolution of scaffold strength in in vivo conditions cannot be predicted by in vitro experiments. The CaP layer, adherence of proteins and the new bone ingrowth likely strengthen the scaffolds, whereas the ongoing dissolution weakens the structure. The strength of the scaffolds sintered at 630 °C for 60 min was within the strength of trabecular bone [3] also after a short SBF immersion. In an in vivo study, these scaffolds were implanted in segmental defects in rabbit femurs for 2–8 weeks and showed good bioactivity and strength: the glass degraded and CaP and new bone layers grew along the scaffold surfaces, providing additional strength [60]. 

As the aim of this work was to produce scaffolds with matching porosity to partially crystallized scaffolds used in a previous animal study [53], a more detailed investigation on the role of the furnace conditions on the scaffold properties was not carried out. Based on the laboratory-scale studies, sintering of moderately strong amorphous scaffolds of bioactive glass S53P4 for use in bone surgery applications appears feasible by properly optimizing the sintering parameters. Converting the sintering parameters to full-scale manufacture requires optimizing the time–temperature conditions to be matched with the size of the scaffold and gas flow, among other considerations. However, these results should encourage further studies.

The results support applying glass S53P4 in new hot-worked products: if sintering to the desired degree can be achieved without crystallization, amorphous BAG S53P4 derived products could be processed and sintered, e.g., via robocasting [11]. Still, as crystallization is a dynamic process, the heating rate and other parameters during the thermal treatment, the BAG particle size and the implant size all play a role in successful processing. Thus, more research is needed to optimize and master the manufacturing parameters. Faster heating to the top sintering temperature may allow a longer sintering time before the onset of crystallization. Therefore, careful optimization of the heating steps to the glass transition temperature (for BAG S53P4: 550 °C [44]) before the final sintering may enable having longer sintering times and more consolidation of the implants. If the sintering is carried out in a mold as in this work, other materials than graphite could offer better heat-transferring properties for optimizing the sintering process. In addition, preheating the mold should also be considered for minimizing the crystallization risks during the top sintering temperature.

## 5. Conclusions

The sintering parameters were optimized for BAG S53P4 to achieve scaffolds with amorphous nature but strength suitable for handling in surgical applications. The chosen parameters yielded amorphous scaffolds with approximately 50% porosity that showed compressive strength comparable to cancellous bone (5 MPa). The porosity of the scaffolds was tailored to match that of weaker, partially crystallized S53P4 scaffolds, which have been reported to support 3D bone ingrowth in rabbit femurs. The dissolution study suggested that the scaffolds gradually degrade and that their necks are strengthened by CaP formation in SBF. The in vitro studies verify the earlier reported in vivo findings of the bone regeneration potential of the optimized S53P4 scaffolds in load-bearing bones. The results aid in developing bioactive glass S53P4-based products for new applications.

## Figures and Tables

**Table 1:** Masses of the granules (one specimen) and amorphous scaffolds (two specimens) before and after the 1-week dissolution tests.

Sample and Solution	Mass before (mg)	Mass after (mg)	Mass Loss (%)
Granules in Tris	233	2	99
Granules in SBF	233	111	52
Scaffolds in Tris	272	72	71
	277	79	74
Scaffolds in SBF	266	128	38
	273	164	53

**Table 2:** Compressive strength of dry scaffolds after sintering and of wet scaffolds after 5 min immersion in SBF. The markings amorphous and crystallized refer to scaffolds sintered for 630 °C for 60 min and 720 °C for 90 min, respectively.

Scaffold and Condition	Compressive Strength (MPa)
amorphous, dry	4.8 ± 0.6
amorphous, wet	2.9 ± 0.5
partially crystallized, dry	1.3 ± 0.1
partially crystallized, wet	1.1 ± 0.2

## Data Availability

All relevant data are shown within the manuscript. Further data are available on request from the corresponding authors.
